# A Practical Compendium of Midwifery, Being the Course of Lectures on Midwifery and on the Diseases of Women and Infants, Delivered at St. Bartholomew’s Hospital, by the Late Robert Gooch, M. D.

**Published:** 1832

**Authors:** 


ART. VII. *A Practical Compendium of Widwifery, being the course of Lectures on Midwifery and on the Diseases of Women and Infants, delivered at St. Bartholomew's Hospital, by the late* ROBERT GOOCH, M. D. Prepared for publication by George Skinner. Philadelphia: Carey and Hart—pp. 120, octavo—1832.

In the last number, we gave an account of a work on some of the Diseases of Women, by Dr. Gooch. The treatise now under consideration was taken from notes of Dr. G's Lectures, and published after his death. It of course wants the finish of the author's hand, and the interest which none but an author himself can give to his conceptions. As it abounds, however, in valuable and original suggestions, it will be found a useful book of reference, and we shall therefore proceed to give some account of the most interesting parts of it to our readers.

LECTURE I. *On the Natural Functions, and on the Diseases of the Female Organs of Generative—Chlorosis.* The indications of cure laid down, are, 1. To improve the condition of the digestive organs; 2. To palliate symptoms; 3. To excite the sexual organs by local stimulants.

The general principles upon which the two first indications are to be conducted do not differ from those laid down by authors. Some remarks upon the use of digitalis will be found on a preceding page. The third indication to be fulfilled by specific remedies he holds in reserve, if the general remedies fail in restoring the discharge—madder, black hellebore, electricity, and marriage. This principle we consider of very great practical importance, not in the use of emmenagogues, but of all medicines from which either a *specific* or an *alterative* effect is expected. It is generally acted upon by the profession where an inflammatory condition prevails, but all are not so fully aware of its importance when the system is in an opposite state.

*Amenorrhœa*, the author remarks, may generally be traced to mental emotion, or to the effects of cold. Of the influence of mental emotion he gives the following curious exemplification.

"I was prescribing for a French lady laboring under a suppression of the menses, and on questioning her, found that she had not menstruated since the Cossacks entered Paris; and it was a well authenticated circumstance, that at the time the allied armies entered Paris, from a similar mental emotion, a great number of French ladies were thus affected. The French Journals of the day contained long lists of patients laboring under this disease. The effect of cold on the uterus during menstruation, or about the time of its occurrence, is a matter of familiar experience; and it is to this influence that the suppression of the menses is, perhaps, the most frequently to be imputed."

The most frequent cause, however, is exposure to cold during or about the time of menstruation. The symptoms, when the disease arises from this cause, may be inflammatory or otherwise. When a phlogistic state of the system prevails, the principles of treatment are obvious; in the opposite condition the following plan of treatment possesses some degree of novelty, and is worthy of a trial.

"But supposing a suppression of this kind occurs in one of the other class of females; here we shall obtain more benefit from that which is termed the antispasmodic, than from the antiphlogistic plan; but in this case it is proper to purge, and that smartly. Put the patient into the warm hip bath, administer diluent drinks, and give antispasmodics, such as five grains of ipecac. comp. and three grains of camphor, every four hours, until all the symptoms subside. The injection of antispasmodic remedies, such as laudanum, or asafœtida, into the rectum, will sometimes act like a charm,—in the latter I have great faith; let two drachms of asafœtida be rubbed down with the yolk of an egg, and mixed with six ounces of warm water; this mixture should be gradually injected up the rectum, which being done, a napkin should be closely applied to the part, and every means made use of in order to restore its retention. If you use laudanum, let the vehicle be small in quantity; an injection of this kind may consist of fifty or sixty drops of laudanum in three ounces of water. It is a great error to suppose that the benefit is to be in proportion to the quantity of fluid injected; this may be true to some extent of purging injections, but the quantity of those of the anodyne description should be small, both because the active ingredient is less diluted, and the injection is the more likely to be retained. The spasmodic affection having yielded, and the nervous irritation being allayed, menstruation generally ensues. But it may so happen, that when the state of spasm has ceased in one variety of the disease, and that of inflammation in the other, the menstrual discharge may still not supervene. Its recurrence may then be expected at the next period; but if it should be suspended month after month, and year after year, this state is distintinguished as that of obstructed menstruation."

*Dysmenorrhœa* is a much more frequent disease than most physicians imagine. It is in the majority of cases accompanied with the general appearance of good health, and therefore attracts less attention from the friends of the patient than its influence upon her happiness requires. Dr. G. thinks it is the principal cause of sterility among young married women. He assigns three causes of the disease; 1. The first and most common is a feeble, nervous, and irritable constitution; 2. Plethora, characterized by a red face and full strong pulse; 3. A disordered condition of the digestive organs or disease of the abdominal viscera.

"In the treatment of this disease there are principally two indications; the first, to alleviate the pain during the menstrual period: and the second, to employ between the intervals of menstruation such remedies as will prevent its recurrence. The first intention is easily accomplished; but the second, if at all, with great difficulty. If the uterus is tender on pressure, with a hot skin, rapid pulse, &c., the existence of an inflammatory state of the uterus is denoted. In this case the hip bath must be used, abstinence from animal food and fermented liquors must be directed, together with a vegetable diet, diluents and purgative medicines; and blood must be abstracted, if the inflammatory state is such as to require it. By these means the pain will be diminished, and the uterus will be disposed to a more healthy action.

In females of a weak, nervous constitution, gentle laxatives, together with anodynes, may be given with advantage on the first occurrence of the symptoms: the use of the hip bath may also be extremely beneficial.

In order to prevent the recurrence of dysmenorrhœa, if the abdominal viscera are disordered, you must endeavor, by a treatment upon general principles, to restore them to a healthy state. If plethora be the cause, you must reduce the quantity of the circulating fluid by a vegetable diet, saline laxatives, and by the abstraction of blood about once a month. If there is debility, you must strengthen the system by the metallic tonics, as by steel, and sulphuric acid combined with the sulphate of zinc; by pure air and gentle exercise;—these are the principle indications of treatment."

As the cure is to be conducted upon general principles, camphor, the volatile tincture of guiacum, and other medicines of this class are to be given only when the system is torpid and inactive.

In all these affections spinal irritation will be found to exist in a greater or less extent. They are diseases of function, not of structure, and the appearances of plethora or debility which may be present, are to be considered as accidental accompaniments, demanding attention, but not presenting indications of radical cure.

*Menorrhagia* is divided by the author into acute and chronic; to which he adds a form of the disease depending upon spasm and irritation. That which depends upon plethora is accompanied by permanent pain, sense of weight, tenderness in the region of the uterus, a hot skin, and a hard febrile pulse; the spasmodic form of the disease is attended with periodical pains resembling the throes of labor; and a quick, contracted, and irritable pulse. It is difficult to make an accurate distinction between these two forms of the disease, probably they are not to be separated in practice. The treatment is to be modified according as one form or the other predominates. If it be inflammatory, by rest, recumbent posture, saline laxatives, bleeding, nitre, digitalis, ipecacuan. in nauseating doses—if attended by irritation, by ipecacuanha in nauseating doses, and antispasmodics, opium or asafœtida, injected into the rectum. There is a close connexion between the pain and the discharge, and if you relieve the former the latter will soon cease.

The chronic form is to be treated by a nutritive diet, gentle laxatives, tonic medicines, acids, bark, steel, and aromatics, but the remedy which Dr. G. especially recommends, is, injections of cold water.

"In using this remedy, I would advise you to act with caution when the weather is very cold. At first take the chill off the water, and by degrees use it cold; but do not use it in the inflammatory or acute form of the complaint, or in that of the spasmodic and irritable kind, for in these it will be productive of mischief. If you use it in the description of cases to which I have alluded, I do not know so good a remedy; but do not suppose it will cure others which are not of the same nature. * * * In administering an enemata, it must not be propelled with great violence, for the gut does not like to be thus assaulted, and will immediately reject it. Our object is that the injection should be retained; therefore it must be given when the patient is in bed. It must be pressed up gradually and gently, at first every night, and then every night and morning. The patient must remain on a sofa during the day, as the recumbent position is the most favorable. If it should be necessary to continue this remedy a long time, we must suspend the employment of it for a few days at the accustomed return of the menstrual period, and afterwards resume it. The benefit obtained from it is sometimes immediate; but if the case should prove tedious, the disease will nevertheless, in general, be ultimately cured by it."

In many cases of menorrhagia we have found aloetic purgatives a very valuable remedy, and if there be no idiosyncrasy to prevent their use, we invariably prescribe them.

This disease, whether it be a simple increase of the menstrual discharge, or a real hæmorrhage, is a disease of function, and consequently, like other affections of the uterus, produces symptoms which cannot be accounted for upon any general principles, whether of inflammation, irritation, or debility. It should also be at all times borne in mind, that what is supposed to be menorrhagia, may sometimes depend upon polypus, abortion, or cancer uteri.

*Leucorrhœa* sometimes proceeds from the vagina, more rarely from the uterus. In this, as in other affections of the uterus, the digestive organs and the general health very frequently, perhaps generally, sympathise. It has been very strenuously disputed whether the local affection stands in the relation of cause or effect. To us it appears evident from the nature of the general symptoms, that they are dependent upon the local affection as their cause, and indeed in a majority of cases the priority of the local affection and their evident dependence upon local irritation, decide this question.

The treatment is naturally dissected to three prominent indications; to improve the general health; to regulate the digestive organs; to suppress the discharge. This last may be attempted either by remedies exerting a specific operation upon the urinary organs, or by astringents. Upon the use of the latter the author makes the following remarks.

"Half a drachm of the sulphate of zinc, dissolved in six ounces of rose water, forms a good injection, some of which may be thrown up the vagina with a syringe twice or three times a day. But it is to be remembered that the best lotion will lose its effects in a week; have then half a dozen; and when one fails, use another for a week, until you have tried them all, and then employ them again. The treatment of leucorrhœa is to a great extent empirical. Cold astringents among the rational practitioners, are in the most general use; but tepid ones are often equally beneficial. Practitioners have exhausted all the cold astringent remedies, and then having recourse to tepid ones, the patient has been cured immediately. The liquor plumbi subacet. dilut. is now used at the Middlesex Hospital, tepid, and with general success. The strength of this application is to be gradually increased until it is doubled. It is in this complaint as in ophthalmia, that you cannot tell *a priori* whether cold or tepid applications will be the most successful; if therefore one fails, the other should be tried."

*Procidentia Uteri* occurs generally, though not invariably, from imprudent conduct after parturition. No disease is more frequently overlooked and is productive in consequence of more distress than this.

"Many cases of imperfect procidentia have been mistaken for those merely of constitutional derangement. The stomach is sympathetically affected by the state of the uterus, and nervous affections and debility ensue, which have been treated with tonics, &c.; but when procidentia has been ascertained, if the uterus is properly managed, the constitution will take care of itself, and these symptoms will speedily disappear. How are we to discover the existence of this complaint? When the uterus protrudes externally between the labia, the patient herself will tell you that she has a falling down of the womb.

If it is an imperfect procidentia, we suspect it from the pain which the patient experiences at the sides of the sacrum, from a sense of painful dragging in the region of the uterus, and of fulness and pressure in the vagina as if something was about to come away. If the patient assumes the horizontal position, these symptoms subside altogether, or are gradually relieved. The uterus presses both behind and before, occasioning a difficulty in passing both urine and fæces. These are the local symptoms. The constitutional symptoms are a disordered state of the stomach and of the whole system, accompanied with a train of nervous sensations."

One remark only of the author we deem it important to mention, and this from experience we know to be highly valuable, which is, whether using a pessary or not, to keep a sponge frequently wet with some astringent solution constantly applied. A roll of muslin of sufficient size to support the uterus, and properly secured by a T bandage, will frequently do more to relieve the disease in two or three days, than the use of astringent washes for a month.

*Chronic Inflammation of the Uterus.*

"Acute inflammation of the uterus, unconnected with pregnancy, is a rare disease: but its occurrence soon after delivery is frequent. Chronic inflammation of this organ, in the unimpregnated state, is very common. The patient complains of a fixed pain in the region of the uterus, aggravated by motion. Pressure above the pubes occasions pain. Pain is felt also at the extremities of the round ligaments. Pain is experienced on touching the cervix uteri, in an examination per vaginam. With these symptoms there is loss of appetite and a disordered state of the alimentary canal. Chronic inflammation of the uterus frequently succeeds abortion; for women do not attach the same importance to this as to labor at the full period of pregnancy, but get up in two or three days and walk about. In ten or fourteen days after this exertion, they sometimes begin to experience the above symptoms. This complaint is also sometimes owing to the insufficient clothing of our English females; and sometimes to a disordered function of the digestive organs. But it is often difficult to trace the disease to its cause: for it is allowed to exist week after week, and a month or two may, perhaps, elapse before we are consulted.

I have no doubt that dysmenorrhœa is frequently owing to chronic inflammation of the uterus. The aching pain in the region of the uterus then continues from one menstrual period to another, and is aggravated during the discharge. From this view of its pathology, the treatment just recommended for chronic inflammation of the uterus would be equally proper in the form of dysmenorrhœa."

LECTURE II. *On Pregnancy.* We pass over the author's remarks on *Conception; The Gravid Uterus; The Signs of Pregnancy*, and come to the *Diseases of Pregnancy.* During pregnancy, the head, chest, stomach, liver, bowels, in fact, every organ are liable to derangements. *The affections of the head* are headach, throbbing, giddiness, &c. These symptoms may depend either upon an inflammatory condition of the system, or upon derangement of the digestive organs. We decide upon the former, by the general indications of the plethora, a florid countenance, full habit, and strong pulse. It is relieved by bleeding, purging, and spare diet. When these affections are severe, they should be carefully watched, as they sometimes terminate in puerperal convulsions. When these symptoms depend upon visceral derangement, they are relieved by active purgatives of such a character, as at once to evacuate the bowels, and give a healthy character to their secretions.

Pregnant women frequently suffer from nervous irritation, with palpitations of the heart, dyspnœa. These affections are frequently relieved by bloodletting and other antiphlogistic means, but they more often depend upon indigestion and acidity, by which they are always aggravated, whatever may be their cause. After the more important indications are attended to, antispasmodics may sometimes relieve the irritation.

*The irritability of the stomach* in pregnant women is sometimes very troublesome. When it resists the usual mode of treatment, the following plan may be worth trying:

"If the irritability of the stomach is very obstinate and distressing, give no nourishment at all at this time for twelve hours; if the mouth is dry, moisten it with a little cold water, afterwards allow a small quantity of food, perhaps some weak chicken broth; prohibit the taking of food again during the next six hours, and direct the patient to drink once at about the middle of this period. Many cases which have resisted all other means, have been much relieved by this treatment. Dr. Vaughan speaks of the powerful influence of opium and starvation, when properly regulated. A lady had taken various remedies without benefit, and it was thought the vomiting was now kept up by habit. It was, therefore, proposed that she should abstain from all food four and twenty hours, at which time her appetite returned, the vomiting ceased, and she recovered. Opium is not requisite in all cases of irritable stomach; but in those of unusual obstinacy, it may be given with advantage. Keep the stomach empty ten or twelve, and let the patient take half a grain of opium every six, hours. In some cases of this description there is a hot skin, a rapid circulation, with pain and tenderness about the præcordia; these symptoms require blood-letting, as from the arm to the amount of eight ounces, or by the application of a dozen leeches to the pit of the stomach. The abstraction of blood greatly relieves this sub-inflammatory or plethoric state of the stomach: mild saline aperients are also to be directed, together with the recumbent position. In sea-sickness it is a common experience, that persons who are immediately sick on sitting or standing upright, are perfectly free from this distressing affection, so long as the recumbent posture is strictly preserved."

*Pain in the hypochondrium*, generally in the right, is sometimes very troublesome. It is slight in the morning, becoming more severe in the course of the afternoon. It probably depends upon the condition of the liver, as it is best relieved by those purgatives which make a decided impression upon the secretions of this organ, such as calomel, and the comp. extract of colocynth—blue pill and aloes.

*The motion of the child* is sometimes so troublesome as to deprive the patient of sleep. It is generally relieved by small bleedings, gentle aperients, and opiates—although they often fail.

We shall take the liberty of quoting the author's remarks upon the œdema and varicose veins, which occur during pregnancy.

"*Œdema* of the lower extremities is likely to occur without any hydropic affection in any other part of the body, at the latter period of pregnancy. It is first remarked towards night about the ankles, by degrees the swelling rises higher, and the legs become of a very large size. The gravid uterus, by its pressure, both obstructs the return of venous blood from the lower extremities, and also compresses the absorbents. The patient goes to bed with her legs swollen; towards morning her face swells, and the swelling of the extremities disappears, but returns as the day advances. Sometimes the œdema is but trifling; at other times it extends not only throughout the lower extremities, but the labia pudendi are enlarged to nearly the size of the head of the child. When labor comes on, the external parts being in this state, the acoucheur, mistaking this swelling for the child's head, may suppose so much of the labor to be over. When first in practice I made this mistake. I was called to a labor; the pains were severe: on examination I found a large tumor between the patient's thighs; and supposing it to be the head of the child, I requested the nurse to get every thing ready. Pain succeeded pain, but there was no advance of the shoulders. I then began to examine more minutely, and found the tumor to consist of an immense enlargement of one of the labia. Had I been more attentive at first, I should have discovered, as I did afterwards, that the skin on the outer side of the tumor was continuous with that of the upper part of the thigh, while the opposite side of the tumor only conducted the finger into the vagina. The labia may be thus enlarged without offering any considerable impediment to the delivery. In general no treatment is required; but if before labor comes on the swelling occasions much pain, I would let out the effused serum by a puncture with a lancet. A lady had both labia greatly swollen and very painful; she refused to have them punctured unless I made use of my eyes, and the tumor which presented itself absolutely astonished me. In half an hour after an opening had been made into the labia, the bed was drenched with serum, and the patient was perfectly easy. I have heard of these punctures exciting inflammation and producing gangrenous ulcers difficult to heal, as scarifications in anasarcous legs sometimes do; but I have never met with an instance of the kind myself, and am rather sceptical in respect to its having occurred.

*Varicose veins* of the lower extremities are very common during pregnancy. This state of the veins arises from the pressure of the enlarged uterus on their trunks, by which the return of blood to the heart is impeded. The remedies for this state of the veins are pressure, cold applications, and the recumbent posture. Pressure should be employed according to Mr. Baynton's method. First strap the leg with adhesive plaster, then apply over this a roller bandage with a moderate degree of tightness, and keep this bandage wet with common or with Goulard water. Practitioners are generally cautious in the employment of pressure, lest in consequence of a compression of the superficial veins of the legs, congestion should occur elsewhere. To prevent this you must, in conjunction with pressure, take away a few ounces of blood, and direct an abstemious diet, at the same time keeping the bowels relaxed by aperients."

Dr. G. devotes a considerable chapter to retroversio uteri, but as this subject has been very ably treated by our countryman, Dr. Dewees, whose works are in the hands of every American practitioner, we deem it unnecessary to refer to it.

Abortion, when it does not arise from accident, but depends upon some constitutional cause, is likely to occur in two opposite conditions of the system, in the plethoric, and in the feeble, nervous, and delicate. Whenever it is threatened, the woman should be separated from the husband, and the general health be very strictly attended to. Dr. G. says, that women subject to dysmenorrhœa generally miscarry: We think it probable that the nervous symptoms which so generally attend on women liable to abortion, proceed, almost always, from some derangement of the uterine function. In guarding against future failures, the condition of this organ should be very minutely inquired into. Women are naturally solicitous about this discharge, when it is entirely suspended, but they frequently permit such deviations from its healthy character, as to produce very considerable distress, and ill health to pass unnoticed. We have frequently seen patients subjected to severe courses of medical treatment for disease of the heart, liver complaint, dyspepsia, &c. when the whole disease originated in some change in the quantity or quality of the menstrual discharge which the patient had never thought it worth while to mention, nor the physician to inquire into. If dysmenorrhœa, profuse or scanty menstruation, fluor albus, and procidentia uteri, were made the subject of closer attention, I am confident that we should hear much less of habitual abortion.

Lecture III. *Of Difficult Labor.* The author divides difficult labor into, 1. Impeded labor; 2. Unnatural presentations; 3. Complicated Labor.

Among the causes of impeded labor, Dr. G. mentions irregular action of the uterus. The pains appear to be active, and the os uteri dilatable, but still the child does not advance. These are called spurious pains, and are generally recommended to be allayed by opium, but Dr. G. asserts that he has rarely given opium with benefit, as it will seldom quiet the pains sufficiently to permit the patient to sleep. Nothing is so valuable as patience, and every resource is to be put in requisition to keep up the spirits of the patient.

The following language respecting ergot will sound strange to the American practitioner. It is due, however, to the author to state, that it is some years since these lectures were delivered. The editor has not the same apology to offer.

"The Americans recommend the ergot of rye, in doses of half a drachm or two scruples, and affirm that the uterus was almost immediately excited by it to a vigorous action. I never used it neither do I credit what has been said respecting its efficacy."

To which the editor appends the following note.

"I have had many opportunities of putting this remedy *fairly* to the test, by administering it in cases, the tediousness of which I had been led to anticipate, by my attendance on the same woman in former labors, which were exceedingly protracted from a sluggish and ineffectual action of the uterus. I have repeatedly given the ergot in half drachm, and two scruple doses; I have given it both in powder and in infusion; and I never witnessed in any one instance the slightest benefit from it."

Dr. G. speaks of a peculiar condition of the os uteri as a cause of tedious labor—instead of being preternaturally hard, it is soft, flabby, and œdematous. This condition is generally produced by the too early rupture of the membranes, in consequence of which the uterus is compressed between the head of the child and the pubes, the circulation is obstructed, and œdema follows. He recommends pressing up the uteri during the pain, and sustaining it in that position during the interval,

"In the thick, spongy, and œdematous state of the os uteri, this plan answers very well; I have found it succeed iu many cases, and there is no occasion for bleeding. Do not suppose that every slowly dilating os uteri is to be thus treated; the delay may be for the reason, that the uterus is acting in a wrong direction, when this method is of no use; or the os uteri may be rigid, dry, hot and painful, when any attempt on your part to dilate it would do mischief; it is only in the thick, spongy, œdematous state of the os uteri that this kind of artificial dilatation can be of service. Pare your nails close, that you may not scratch the os uteri, and do not press it upwards, and against the symphysis pubis, by which you may irritate or injure the urethra, but rather on one side of the course of the urethra. The os uteri is sometimes thus slipped over the head with the greatest facility, and I have been surprised at the advantage I have gained by it; but if this cannot be accomplished in ten or a dozen pains, you will not succeed at all, and you had better not persevere in the attempt."

The author is very full in his remarks upon the consequences of protracted labor, and in his directions for the use of instruments. The following curious history respecting the invention of the forceps is worth relating:

"The first account we have of the forceps was in the year 1743; and these instruments were supposed to be the same as those which

Chamberlain had employed, the truth of which conjecture was not confirmed until within the last four or five years. The history which I received from Mr. Carwardine, of Essex, runs thus;—Woodham Mortimer Hall, in Essex, is an old manor house, and was once the residence of old Chamberlain, since whose time it has been occupied by several individuals. About four years since, Mr. Kemble, in exploring an old closet in this house, discovered a trap-door, which being opened, was found to lead into another closet, in which were boxes containing letters, rings, jewels, trinkets, among other things, old Chamberlain's will, and a ring with the hair of Mrs. Chamberlain, which appeared to have been placed there by her daughter, with the following written memorandum:—"This lock of hair was taken from my dear mother after her death." There were also two bags of instruments, consisting of different specimens of the forceps and vectis: there was a petition also to the House of Commons, lamenting the number of deaths occasioned by ignorant midwives, and stating that himself and sons possessed a knowledge of means by which women may be delivered without injury to either the mother or child; and hinting slyly, that if the House of Commons would grant him a sum of money, he would, at their command, disclose the secret. Old Chamberlain was buried in the church yard near by; and on his tomb stone is engraved "that he was born in 1601, and died in 1692, and had been physician to three kings; Charles the First, Charles the Second, and James the Second; that he was baptised when he was 48 years of age, after which he kept the Sabbath." These circumstances leave no doubt that the instruments found in the closet were those which Chamberlain employed, which were similar to the forceps and vectis now in use, and of the application of which I have next to speak."

The author disapproves very much of the use of the crotchet, and recommends a substitute.

"It is a diabolical instrument: even in the hands of the best practitioner it is a dangerous one. I never myself met with any accident in the use of it, not because it did not sometimes slip, but because I chose rather to lacerate my hand than my patient's vagina. I shall never use it again; but instead of it, an instrument somewhat resembling lithotomy forceps, rather larger and longer, with teeth at the extremity of each blade, by which the hold is rendered very firm. You pass it closed up the vagina, and when it arrives at the opening in the skull, the handles are separated; one blade is introduced within the opening, the other is on the outside, and closing the handles you grasp a portion of the skull. The extraction is to be made carefully, at first downwards and backwards; but when the head presses on the perinæum, downwards and forwards, if the bone which is grasped by the forceps gives way, extract it, and grasp another portion. This is an old instrument, the invention of which is claimed by Lamotte."

Dr. G. devotes two pages to the subject of ARTIFICIAL PREMATURE LABOR, and describes two modes of performing the operation; one by evacuating the liquor amnii—the second we shall leave him to describe in his own words, together with his views respecting the advantages and disadvantages of the operation.

"The second method of inducing premature labor is to pass the hand completely into the vagina, and introducing your fore-finger gradually through the os uteri, peel off the membranes all round the cervical portion of the uterus; after which it is found that the expulsive action of the uterus will generally come on in about forty-eight hours. The advantage of this method is that you have the unruptured membranes to dilate the os uteri, and the liquor amnii to defend the head of the child. If premature labor does not come on in two or three days from the separation of the membranes from the neck of the uterus, you must have recourse to the first-mentioned operation of puncturing them.

Foreigners are exceedingly afraid of this operation; and certain it is that great disturbance of the nervous system is produced by it; severe rigors, rapid pulse and delirium are the occasional consequences, but these symptoms, proceeding from nervous irritation, do not continue long enough to produce any serious consequences: they sometimes cease as soon as the pains commence; and if not then, they cease after the uterus is emptied. About nineteen children have been thus prematurely born, and afterwards reared in this country. This practice, therefore, gives a chance of preserving the life both of the mother and child."

Of the author's remarks upon preternatural presentations, we shall only notice those upon *presentation of the arm.* When this presentation is ascertained, the operation of turning is to be performed as soon as it can be resorted to without risk. When the os uteri is somewhat dilated and dilatable, the pains are to be suppressed by venesection, if the circumstances of the patient will at all admit of it, or at all events, with a full opiate—70 or 80 drops of laudanum. When the uterine effort has subsided, the hand is to be introduced—the right or left, according to the position of the child—in such a manner as that the palm of the hand will correspond to the abdomen of the child, that the feet may be more readily grasped and brought down. The position of the child will be determined by that of its hand.

"The rule, then, for the employment of either hand in the operation of turning, is this:—If the palm of the child's hand is towards the abdomen of the mother, you are to pass up your right hand into the anterior part of the uterus; if the back of the child's hand is towards the belly of the mother, you are to pass up your left hand into the posterior part of the uterus. In every turning case you are to introduce your hand by the side of the presenting part; as the feet are brought down this goes up. If both feet cannot be readily grasped and brought down together, bring down one first, and then the other; and in the progress of extraction, remember to attend to the direction of the axis of the pelvis. Dr. Thynne was called to a case in which the attending practitioner could not turn. The failure arose from his having attempted the operation only with his right hand, when the back of the child's hand was towards the mother's belly. Dr. Thynne introduced his left hand, and turned the child with facility."

The following quotation gives, probably, the true explanation of the manner in which the arm presentation generally takes place, and also of the phenomenon which Dr. Denman has called the *spontaneous evolution of the fœtus.*

"Sometimes when the hand or shoulder presents, the head rests on the edge of the brim of the pelvis; and if you return the presenting part, the uterus is so stimulated to vigorous action, by the introduction of the hand, that the head is thrown off the brim of the pelvis, and descends as in a natural presentation. I have succeeded in this way in many cases, and in some to which I was called for the express purpose of turning; while apparently only making a common examination, I have returned the presenting part, and then waited for a pain, which has brought down the head. I have then desired the attending practitioner to examine, and he has been surprised to find that the arm was converted into a head presentation. On one occasion when the arm presented I pushed it up, and down came the funis, which I carefully replaced; the uterus immediately contracting forced down the head, which of course continued to be the presenting part until it was expelled. In arm presentations when the body of the child is completely across the pelvis, you must turn and deliver; but if the head is only a little removed from its natural position, then return the hand, in the hope that the head at the next pain, will assume its place. Two queries here suggest themselves: the first, If when the head is but little removed from its natural position, the labor were allowed to proceed without interference, would not the position of the child become transverse relatively to the pelvis, as the arm was forced lower into the vagina? The second, if an arm presentation were detected in a very early stage of the labor, would it not be found that the head was always a little removed from its customary position?

It has been asserted by some writers when the arm at first presents that this recedes, and the breech takes its place; this is called a spontaneous evolution. Dr. Denman stated that the breech is made to descend, and at the same time the arm goes up, by the continued action of the uterus. In proof of this fact, he published several cases, together with the inference that arm presentations may be entirely left to nature; this conclusion was, however, invalidated by his subsequent experience. Dr. Douglass denies this change of position altogether; he observes, that when the arm and shoulder were pushed under the arch of the pubes, additional space is gained at the back part of the pelvis, the breech is forced down, distends the perinæum, and the child is born double. This I consider the most correct explanation."

Of complicated labor, one of the most formidable complications that the practitioner has to contend with, is that of *puerperal convulsions*. Dr. G. remarks that there are two classes peculiarly liable to this affection—the plethoric and the irritable. Depressing passions of the mind are very liable to produce it—and hence in lying-in hospitals, a very great proportion of the cases are among unmarried women. Bleeding is the sheet anchor of the practitioner in the estimation of the author, without regard to the peculiarities of the constitution in which it occurs. Neither does he appear to attach the importance to the delivery of the patient that most practitioners do; although he appears to be inclined to recommend, after a sufficient degree of depletion, provided the os uteri is sufficiently relaxed to permit it to be done without violence.

The practice of Dr. Dewees appears to be sanctioned both by experience and sound judgment, namely, to look upon delivery as an important object, always to be kept in view, but not to be attempted until the oppression of the brain, and the rigidity of the os uteri is overcome by venesection. A sufficient degree of bloodletting to relieve the brain will render the os uteri sufficiently dilatable to make turning practicable and easy; but before this, the attempt will only increase the irritation which it is the object of delivery to get rid of.

LECTURE IV. *On the general Management of Women after Delivery.* Of the articles alluded to in this chapter, we shall only notice his remarks on milk abscess. The following rule of practice appears to us highly reasonable.

"The treatment, to some extent, resembles that of phlegmonous inflammation in any other part: it consists in the application of leeches, in purging, and a low, cooling diet; in addition to which, it is of the highest importance to keep the lactiferous tubes empty. These minute vessels are, I think, spasmodically affected; be this as it may, the breasts are most effectually disposed to permit the escape of the milk by hot fomentations, which are to be applied, for half an hour at a time, by means of flannels wrung out of hot water, and renewed every five minutes, while the escape of the steam is prevented by placing over them a wooden bowl of sufficient size, or some such contrivance. After the fomentation, immediately apply the child to the breast, or get it drawn by a woman, when it will be emptied readily, and the patient will be considerably relieved. The fomentations and drawing of the breast should afterwards be so frequently repeated as to prevent an unpleasant degree of distention; in the intervals, cover the breasts with a warm poultice, which I am informed diminishes the secretion of milk. Under a steady perseverence in this plan, the excitement of the constitution is quieted, the hard tumor gradually diminishes and disappears. Some practitioners recommend cold applications, but they are extremely predjudicial, if only by rendering the abstraction of milk more difficult, to say nothing of the well known fact, that the disease is frequently produced by cold."

Dr. G. thinks that although by opening the abscess, when suppuration has taken place, we relieve the present sufferings of the patient, yet we produce a wound more difficult to heal, and therefore he recommends this part of the process to be left to nature, relieving the severity of the pain by opiates given according to the necessity of the case.

This chapter contains a valuable article on phlegmasia dolens. The treatment is not different from that generally recommended. After the inflammatory symptoms subside, he recommends strapping the limb from the toes to the groin with adhesive plaster, a practice which we can recommend with confidence as likely to be followed by the happiest results in reducing the swelling. It has long been asserted that the disease is not confined to puerperal women. Dr. G. has met with an instance occurring after abortion. He thinks there is reason to believe that an unhealthy and irritated state of the uterus, attended with a fetid discharge, may give rise to it, and recommends the vagina to be well cleansed of the lochial or other fetid discharges, by the use of a syringe and warm water. He has seen the disease from the application of a ligature to a polypus uteri, which was followed by a highly offensive discharge.

> "I have sometimes met with a disease very much resembling phlegmasia dolens: with the exception, however, that the limb does not swell; there is considerable pain shooting from the groin down the thigh, which is attended with fever. The constitutional treatment of this affection will consist in the exhibition of purgatives, sudorifics, &c.; and I have found a circular blister round the thigh, just above the knee, like a garter, productive of great benefit."

We gave in the preceding number of this Journal, some account of another work by the author, on the diseases of females. That volume having received the finishing hand of the author, and being devoted to a few select subjects to which he had devoted peculiar attention, possessed a fulness and accuracy of detail, and a finish of style and execution which does not belong to the present volume. It is peculiarly valuable for the graphic interest of the style and the great number of interesting and instructive cases with which the author illustrated the character of the disease, and the practice with which he combatted it. The Compendium of Midwifery labors under the double disadvantage of being a posthumous work, and of being a mere report of Dr. G's Lectures. It will be found, however, a judicious system, will arranged, and full of valuable suggestions.

